# Supplementary material for: Assessing ascertainment bias in atrial fibrillation across US minority groups
Source: PLoS One. 2024 Apr 16;19(4):e0301991. doi: 10.1371/journal.pone.0301991 (PMC11020362; doi:10.1371/journal.pone.0301991)
Supplement: S1 Table — (DOCX) [file pone.0301991.s001.docx]

| **Study** | **Enroll. Period** | **Enrolled Population** | **Diagnosis Method** | **Comments** | **Study type** |
| --- | --- | --- | --- | --- | --- |
| ARIC^27^ | 1987 1989 | 15792 adults  45 to 65 years 4 communities | ECG conducted at baseline and 3 follow-up visits, hospitalizations (ICD-9) and death certificates. | Hospital discharge diagnosis covered 88% of AF cases. Follow up until 2004. | C |
| NOMASS^23^ | 1993 1997 | 1844 adults Northern Manhattan | Self-report. | Stroke cases and controls (2:1). | C |
| ATRIA^34^ | 1996 1997 | Adults 20 years or older Kaiser Permanente Northern California | Hospital discharge diagnosis and billing claims databases for health plan and out-of-network admissions, database of emergency department and outpatient clinic visits (ICD-9). |  | C |
| EPOCH^31^ | 1999 2000 | 1373 adults Kaiser Permanente Northern California | Admission ECG, physician-assigned diagnoses in the medical records and/or the presence of corresponding ICD-9 codes in hospital discharge or ambulatory visit clinical databases during the five years before the index hospitalization. | Heart failure patients. | C |
| SCCS ^32^ | 2002 2009 | 85000 adults 65 year or older Southeastern US | Medical claims with ICD-9. |  | C |
| SES ^28^ | 2000 2013 | 239741 adults New York | Determined by (~4) ECGs. |  | C |
| HSS HEPS TOFMSS ^35^ | 2000 2005 | 6611 participants across 3 studies. | Protocol-driven ECGs  HSS – time of enrolment HERS – time of enrollment and yearly visits TOFMSS – 4–8-hour ECG | 3 combined cohort studies (Heart and Soul, Heart and Estrogen-Progestin and Osteoporotic Fractures in Men Sleep). | C |
| MESA ^25,36^ | 2000 2018 | 6663 adults (C) 1556 adults (M) 45 to 84 years. | Hospital discharge codes  (ICD-9) and monitor. | Asian = Chinese Americans | C M |
| NIS HF ^26^ | 2001 2011 | >95% of the US inpatient population. | Inpatient care database  (ICD-9). | Heart failure patients. | C |
| REGARDS ^24^ | 2003 2007 | 30239 adults  45 year or older 56% in stroke belt. | Self-report and/or study ECG. | REGARDS participants who were Black had a lower odds ratio of being aware of their atrial fibrillation (OR 0.32, 95%CI 0.19-0.54) | C |
| HCUP ^30^ | 2005 2009 | 13967949 adults California | Emergency Department Databases, Inpatient Databases, and Ambulatory Surgery Databases (ICD-9). | Inpatient hospitalization is primary source of diagnoses. | C |
| Medicare 2007 ^37^ | 1993 2007 | Medicare population 65 year and older | Inpatient and outpatient claims  (ICD-9). |  | C |
| California Health System ^29^ | Until 2008 | 430317 adults 60 year and older | ECG archives and databases. | Only abstract available. | C |
| CRIC ^38^ | 2001 2010 | 3267 adults 21 to 74 years | Self-report or ECG at baseline. | CKD patients. | C |
| All of Us ^39^ | 2017 2019 | 173099 adults | Self-report and from  data obtained from EHR  (SNOMED CT). |  | C |
| Pacemaker study ^33^ | 2005 2011 | 101773 adults | Administrative claims data on emergency department visits and acute care hospitalizations. | Patients with a pacemaker, the regular interrogation of which reduces the likelihood of undiagnosed AF. | M |
| ARIC 48h ^47^ | 2014 2016 | 2434 adults | Atrial Fibrillation defined on ambulatory ECG, ARIC study visit ECG, or hospitalization. |  | C M |
