# Supplementary material for: Assessing ascertainment bias in atrial fibrillation across US minority groups
Source: PLoS One. 2024 Apr 16;19(4):e0301991. doi: 10.1371/journal.pone.0301991 (PMC11020362; doi:10.1371/journal.pone.0301991)
Supplement: S2 Table — (DOCX) [file pone.0301991.s002.docx]

| **Study** | **Non-Hispanic White (%)** | **Black  (% - rel. diff.)** | | **Hispanic**  **(% - rel. diff.)** | | **Asian  (% - rel. diff.)** | |
| --- | --- | --- | --- | --- | --- | --- | --- |
| **ARIC**^27^ | 7.9 | 4.8 | -39% | - | - | - | - |
| **NOMASS**^23^ | 8 | 5 | -37% | 4 | -50% | - | - |
| **ATRIA**^34^ | 2.2 | 1.5 | -32% | - | - | - | - |
| **EPOCH**^31^ | 38.3 | 19.7 | -49% | - | - | - | - |
| **SCCS**^32^ | 15 | 11 | -27% | - | - | - | - |
| **SES**^28^ | 16.8 | 8.7 | -48% | 7.7 | -54% | - | - |
| **HSS HEPS TOFMSS**^35^ | 4.47 | 1.26 | -72% | 2.71 | -39% | 2.02 | -55% |
| **MESA (Clinical)**^25^ | 11.3 | 6.6 | -42% | 7.8 | -31% | 9.9 | -12% |
| **NIS HF**^26^ | 40 | 18.8 | -53% | 24.4 | -39% | 32.5 | -19% |
| **REGARDS**^24^ | 8.5 | 6.8 | -20% | - | - | - | - |
| **HCUP**^30^ | 3.4 | 1.8 | -47% | 1.4 | -59% | 2.4 | -30% |
| **Medicare 2007**^37^ | 9.1 | 4.6 | -49% | - | - | - | - |
| **California Health System**^29^ | 8 | 3.8 | -52% | 3.6 | -55% | 3.9 | -51% |
| **CRIC**^38^ | 20.1 | 16.8 | -16% | - | - | - | - |
| **All of Us (EHR)**^39^ | 6.3 | 2.8 | -56% | 2.7 | -57% | 2.1 | -67% |
| **All of Us (Survey)**^39^ | 5.2 | 2.2 | -58% | 1.8 | -65% | 1.8 | -65% |
