# Supplementary material for: Assessing ascertainment bias in atrial fibrillation across US minority groups
Source: PLoS One. 2024 Apr 16;19(4):e0301991. doi: 10.1371/journal.pone.0301991 (PMC11020362; doi:10.1371/journal.pone.0301991)
Supplement: S3 Table — (DOCX) [file pone.0301991.s003.docx]

| **Study** | **Non-Hispanic White (%)** | **Black  (% - rel. diff.)** | | **Hispanic**  **(% - rel. diff.)** | | **Asian  (% - rel. diff.)** | |
| --- | --- | --- | --- | --- | --- | --- | --- |
| MESA (Monitoring)^25^ | 7.1 | 6.4 | -10% | 6.9 | -3% | 5.2 | -27% |
| Pacemaker study^33^ | 25.5 | 21.4 | -16% | - | - | - | - |
| ARIC 48h (Monitoring)^47^ | 5.5 | 3.2 | -42% | - | - | - | - |
