# Supplementary figures and images for: Assessing ascertainment bias in atrial fibrillation across US minority groups
Source: PLoS One. 2024 Apr 16;19(4):e0301991. doi: 10.1371/journal.pone.0301991 (PMC11020362; doi:10.1371/journal.pone.0301991)

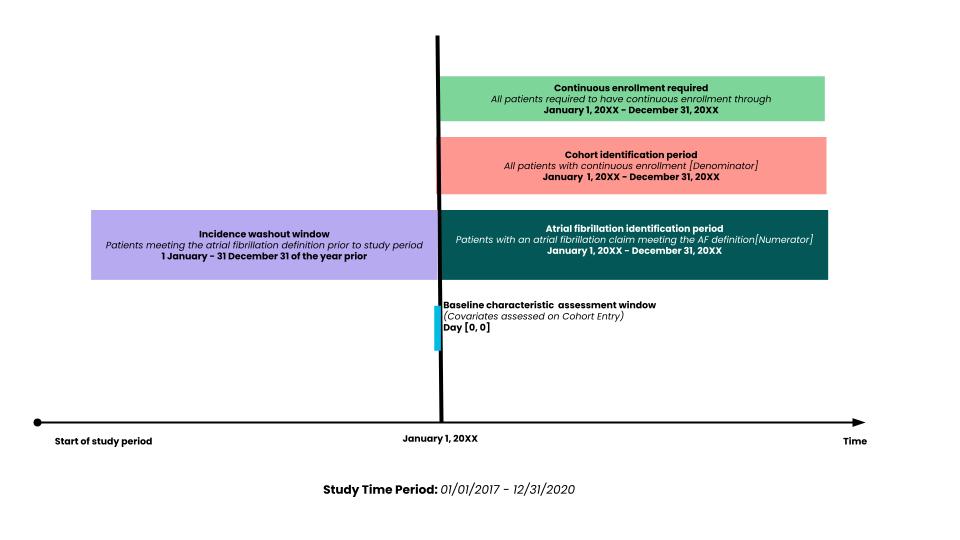

Supplement: S1 Fig — (JPG) [file pone.0301991.s004.jpg]

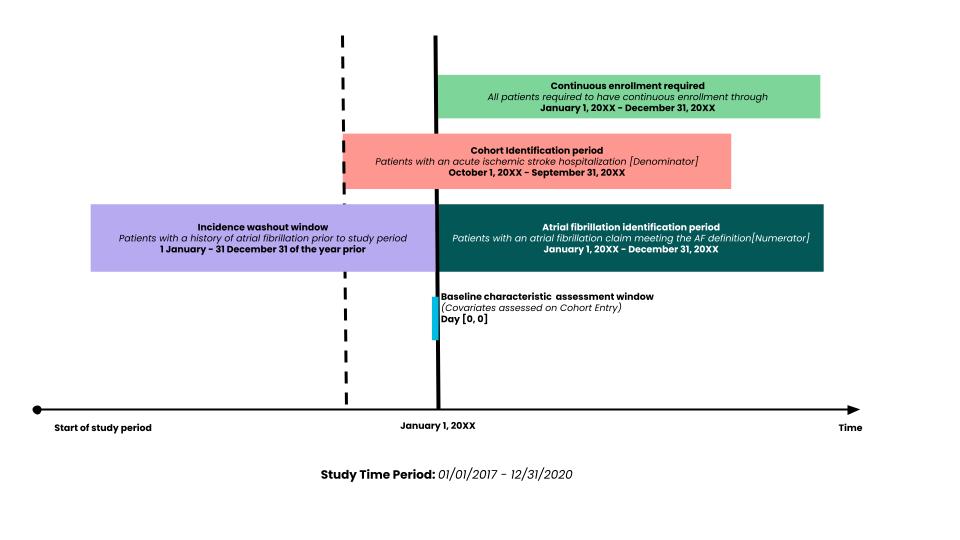

Supplement: S2 Fig — (JPG) [file pone.0301991.s005.jpg]

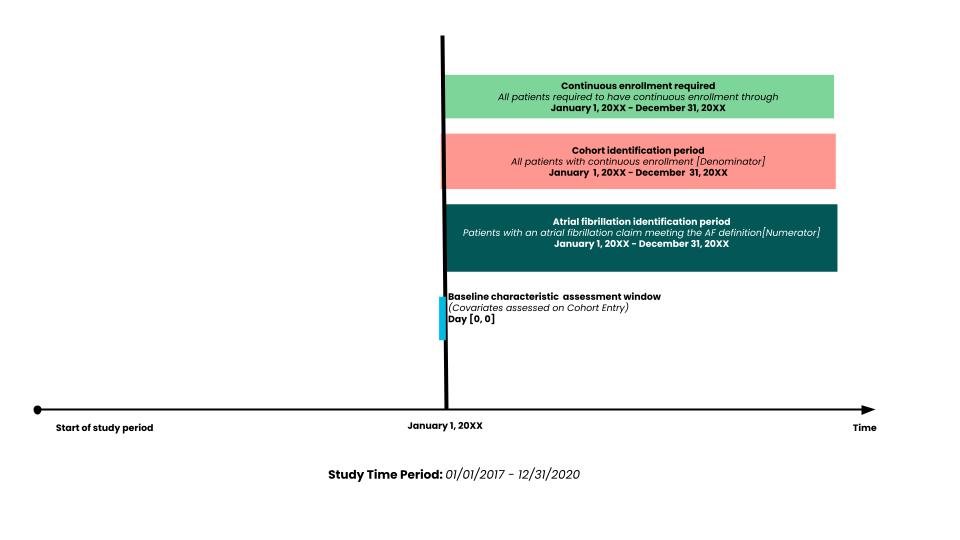

Supplement: S3 Fig — (JPG) [file pone.0301991.s006.jpg]

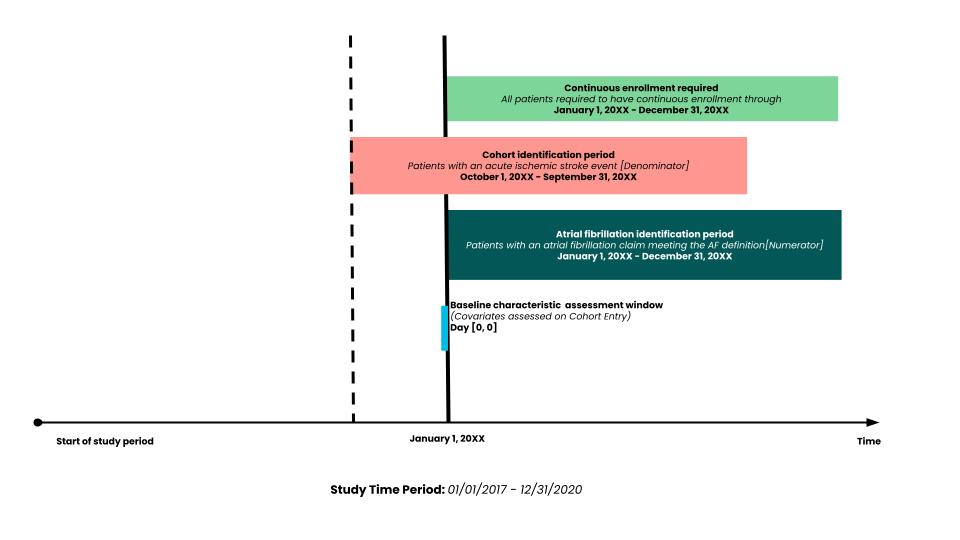

Supplement: S4 Fig — (JPG) [file pone.0301991.s007.jpg]
