# Supplementary material for: Assessing ascertainment bias in atrial fibrillation across US minority groups
Source: PLoS One. 2024 Apr 16;19(4):e0301991. doi: 10.1371/journal.pone.0301991 (PMC11020362; doi:10.1371/journal.pone.0301991)
Supplement: Attachment — Submitted filename: Response to Reviewers.docx [file pone.0301991.s008.docx]

Lars Hulstaert
Johnson & Johnson GmbH

Johnson & Johnson Platz 2
41470 Neuss
Germany
lhulsta1@its.jnj.com

**To: PLOS One**

Dear Editor,

I hope this letter finds you well. I appreciate the time and effort invested by the editorial team and reviewers in evaluating my Research Article titled "Assessing ascertainment bias in atrial fibrillation across US minority groups" for the PLOS One Journal. I am grateful for the feedback provided, and I would like to submit a rebuttal addressing the comments raised during the review process.

**Editor Comments**

| **Comment** | **Response** |
| --- | --- |
| While the study focused on investigating the presence of ascertainment bias, the study's design lends itself to ascertainment bias; in that those patients with a recent stroke would have increased focus on recognizing AF.  I encourage the authors to discuss this oddity and include language or evidence to support the stance that claims data represents real-world data. | Yes, it is possible that patients with AF given a recent ischemic stroke (AF \| stroke & claims) may be different from overall AF population (AF \| claims). In the paper we refer to the [AF \| claims] group as 'clinical-based detection setting' and the [AF \| stroke, claims] group as 'monitoring-based detection setting'. However, since the stroke subgroup is a subset of the overall AF population observed in the claims data (i.e. all the AF stroke patients also met the general inclusion criteria for AF) therefore they should come from the same distribution.  In clinical practice, all patients that have an ischemic stroke event receive AF-monitoring/screening, therefore we control for bias in screening in this population. Further evidence of this bias control is the alignment of our results in claims data with previous cited observational studies, where the bias control is achieved through monitoring-based diagnosis methods.  We updated the paragraph in the limitations section: “This study examines ascertainment bias in AF, and the study design controls for such bias, by contrasting the overall population with a cohort of patients with a recent ischemic stroke, for which AF monitoring is part of stroke guidelines. The study relies on claims data to characterize differences, as opposed to observational studies that rely on active monitoring-based diagnosis methods for AF to assess the impact of ascertainment bias in AF differences across racial and ethnic groups.” |
| Line 111: Ref 27 is repeated. Please edit. | We removed the duplicate reference. |
| Again, in Line 111, I suggest using “blacks” instead of “African Americans” as “blacks” is more encompassing and would be in keeping with the style of the CMD. Alternatively, provide a working definition of your preferred terms. | We aligned the manuscript to the OMB standards (<https://orwh.od.nih.gov/toolkit/other-relevant-federal-policies/OMB-standards>), and modified instances of ‘Black’ or ‘African American’ to ‘Black or African American’ and ‘Hispanic’ to ‘Hispanic or Latino’. To align to recent research standards, we removed racial and ethnic terms in noun form. (https://jamanetwork.com/journals/jama/fullarticle/2783090). We updated the manuscript to reflect both OMB standards and adjectival forms. |
| Line 153: “The patient’s name and geography are...” Are the authors describing the data vendor’s treatment of the claims data or their manipulation of it? Please clarify as the latter situation may be misconstrued as a HIPAA breach. The statement on Line 156 claiming de-identified data must be reconciled with the previous quote. | We adapted the statement to clarify that the data vendor used patient name & geography, not the authors:  “The patient’s name and geography are used by the data vendor to map the patient to one of five race and ethnicity categories…”. |
| Line 170: Please note that including ICD-10 codes such as I64.x (which could include hemorrhagic strokes) will reduce the specificity of your inclusion. One would not routinely monitor for AF in hemorrhagic strokes. Please clarify or include as a limitation in the discussion. | We reviewed the ischemic stroke diagnosis codes based on published literature on coding sensitivity and specificity for stroke and stroke risk factors. We acknowledge the potential for miscoding and subsequent risk for misclassification bias in the limitations section:  “There is the potential for miscoding in the atrial fibrillation and ischemic stroke events and subsequent risk of misclassification bias in patient records due to provider coding patterns (e.g., using diagnosis codes to indicate rule-out criterion) or incorrect coding (e.g., data entry errors), which may lead to misclassification of diagnoses or patient characteristics. The algorithms identified for the current study have been previously validated with a focus on precision [42, 44].” |
| The expression shown on Line 226 is the prevalence ratio (by definition), but it was called “magnitude of difference”, which would invoke the absolute difference. Since this expression is used throughout the paper, it constitutes a major flaw. | We revised the manuscript and opted for ‘prevalence ratio’ as opposed to ‘magnitude of difference’. Prevalence ratio is a more widely recognized term for describing the formula. The formula, the results, and the conclusion of the manuscript remain the same. |

**Reviewer 1 Comments**

| **Comment** | **Response** |
| --- | --- |
| as regard the manuscript "Assessing ascertainment bias in atrial fibrillation across US minority groups type of AF" paroxysmal, persistent or permanent is not included. | The ICD-10 diagnosis codes that were used for atrial fibrillation (I48*) contain paroxysmal (I48.0) and persistent or permanent (I48.1) atrial fibrillation. |
| has bled score and usage of anticoagulants are not mentioned in data | The HAS-BLED score and usage of anticoagulants was not included in the manuscript, in favor of the CHA2DS2-VASc score. The CHA2DS2-VASc score measures Atrial Fibrillation stroke risk, which more closely aligns with the study design. The HAS-BLED score and usage of anticoagulants on the other hand focusses on correctable risk factors for bleeding. Note that both risk scores have a strong overlap in terms of criteria (hypertension, age, stroke & vascular disease). |

I have reviewed each comment, and I believe the revisions strengthened the manuscript.

I would like to express my gratitude for the opportunity to submit this revised manuscript for reconsideration. I believe the revisions adequately address the concerns raised, and I am confident that the manuscript is now in line with the standards and objectives of PLOS One.

Thank you for considering our work for inclusion in your journal. We look forward to your feedback.

Sincerely,

Lars Hulstaert
Johnson & Johnson
